# Extensor Pollicis Longus Rupture After Distal Radius Fracture: Incidence, Associations, and Functional Outcomes in a Prospective Cohort

**DOI:** 10.1016/j.jhsg.2026.101179

**Published:** 2026-11-06

**Authors:** Arkan S. Sayed-Noor, Hasan A. Abo Jouma, Bariq Al-Amiry, Ahmed Alomari, Mats Wadsten

**Affiliations:** ∗ Clinical Sciences Department, College of Medicine, University of Sharjah, United Arab Emirates; † Department of Radiology, Karolinska University Hospital, Stockholm, Sweden; ‡ Department of Diagnostics and Intervention, Umeå University, Sweden

**Keywords:** Complications, Distal radius fracture, Extensor pollicis longus, Patient-reported outcome, Tendon rupture

## Abstract

**Purpose:**

Rupture of the extensor pollicis longus (EPL) tendon is an uncommon but clinically important complication of distal radius fractures. We investigated its incidence, timing, associated factors, and functional consequences in a prospective cohort.

**Methods:**

This is a secondary analysis of a prospective multicenter cohort at two Swedish orthopedic departments, 2009–2012. Consecutive adults with acute distal radius fractures were enrolled and followed to 12 months. EPL function was assessed by active thumb extension testing at 3 and 12 months. Extensor tendon status was recorded in 419 fractures at 3 months and 373 at 12 months. Associations were analyzed between EPL rupture and age, sex, initial fracture displacement, dorsal comminution, treatment modality, surgical technique, 12-month grip strength, Disabilities of the Arm, Shoulder, and Hand (*Quick*DASH), and EQ-5D.

**Results:**

Ten EPL ruptures were identified among the 419 fractures assessed at 3 months, representing an incidence of 2.4%. One rupture was acute; none occurred between 3 and 12 months. Seven ruptures occurred in undisplaced or minimally displaced fractures, and three occurred in displaced fractures (*P* = .06). Four followed surgical treatment, and six followed nonsurgical management. EPL rupture was not significantly associated with age, sex, dorsal comminution, treatment type, or surgical technique. At 12 months, grip strength, *Quick*DASH, and EQ-5D did not differ significantly between patients with and without EPL rupture. Seven of the 10 patients with rupture underwent tendon transfer; 12-month *Quick*DASH scores did not differ between these subgroups.

**Conclusions:**

EPL rupture occurred in 2.4% of distal radius fractures, and all cases occurred within 3 months. No clear demographic, fracture-related, or treatment-related risk factors were identified. Long-term grip strength, function, and quality of life were comparable between patients with and without rupture. Outcomes were acceptable in the few selected patients managed nonsurgically, but the study was not powered to compare treatments. These findings underscore the importance of early surveillance and support an individualized approach.

**Type of study/level of evidence:**

Prognostic II.

Distal radius fractures (DRFs) are among the most frequently seen fractures in orthopedic practice, affecting individuals across all age groups.^1^, ^2^, ^3^ They are particularly common in older adults, often because of osteoporosis, whereas younger patients usually sustain them through high-energy trauma. Advances in surgical techniques and implant design have improved outcomes after DRFs; however, complications still occur.^4^ One complication that can have a marked impact on hand function and quality of life, yet often remains underestimated, is rupture of the extensor pollicis longus (EPL) tendon.^5^, ^6^, ^7^, ^8^, ^9^, ^10^

The EPL tendon is crucial for thumb extension, and its rupture can result in considerable functional deficits.^5^, ^6^, ^7^, ^8^, ^9^, ^10^ This complication was first described in 1876 by Duplay,^11^ with its association with DRFs acknowledged in the early 1930s. Although relatively uncommon—reported in approximately 0.2% to 5% of DRF cases—it is clinically important. The EPL ruptures are broadly classified into acute and delayed types. Acute ruptures are usually caused by direct trauma, such as sharp bone fragments or penetrating injury. In contrast, delayed ruptures evolve progressively because of factors such as vascular compromise, chronic mechanical friction, or increased pressure within the third tendon compartment.^5^

Delayed EPL ruptures can occur unpredictably, sometimes as early as 3 weeks postinjury, or in rare cases, years later. The anatomy around Lister’s tubercle—a dorsal prominence on the radius—plays a major role in this process, given the EPL tendon’s limited blood supply. Fractures involving Lister’s tubercle can further compromise tendon vascularity. Additional risk factors include hematoma formation, fracture callus impingement, and continuous tendon attrition. Microangiographic studies have confirmed a hypovascular zone within the EPL tendon, supporting ischemia as a contributing factor in delayed rupture.^7^, ^8^, ^9^, ^10^

Surgical treatment, particularly open reduction and internal fixation, has also been linked to EPL rupture, with a reported incidence of approximately 0.8%.^10^ This is often attributed to hardware penetrating the dorsal cortex and irritating the tendon.^9^ Fractures involving dorsal comminution or Lister’s tubercle may increase this risk.^8^

Despite the available literature, important knowledge gaps remain regarding the incidence, timing, and risk factors for EPL rupture after DRFs, including the role of fracture characteristics and treatment modality. This study aimed to address these gaps in a prospective cohort of patients with DRFs.

## Material and Methods

This study is a secondary analysis of a prospective, multicenter cohort study performed at the orthopedic departments of two hospitals in Sweden, from 2009 to 2012. Consecutive adult patients presenting with acute DRFs were enrolled. The original cohort was established to assess radiological and clinical outcomes following DRFs and has previously been reported.^12^, ^13^ In the present analysis, we specifically investigated the incidence, timing, outcome, and potential predictors of EPL tendon rupture within this established data set.

Follow-up evaluations occurred at three predefined time points: baseline (day of presentation), 3 months postinjury, and 12 months postinjury.

The initial cohort included 464 DRFs in 451 patients at baseline. Two patients were excluded because they lived outside the hospital’s catchment area, leaving 462 fractures in 449 patients; 13 patients sustained bilateral fractures. Because both the exposures of interest and the outcome are wrist specific, the fracture was used as the unit of analysis.

Tendon integrity was prospectively and explicitly documented by clinical examination (active thumb extension testing) at 3 and 12 months. Patients with intact tendon function at 12 months were also considered intact at 3 months if data at that time point were missing. Thus, EPL data from 419 DRFs were available at 3 months and from 373 at 12 months. Ten EPL ruptures were confirmed, one acute (at presentation) and nine delayed (manifesting within 3 months postinjury). No additional ruptures were detected between 3 and 12 months. At 12 months, grip strength was measured in 373 fractured wrists, and questionnaire data for the *Quick*DASH and EQ-5D were obtained for 380 fractured wrists.^14^, ^15^

Associations between EPL rupture and potential predictors were examined for demographic factors (age and sex), fracture morphology (initial displacement and presence of dorsal comminution), treatment (surgical versus nonsurgical management and surgical subtype), and functional outcomes evaluated with a 12-month *Quick*DASH score, EQ-5D, and handgrip strength using a standardized Jamar dynamometer protocol.^14^, ^15^, ^16^

Categorical comparisons were performed using Fisher exact test; continuous variables were analyzed using independent *t* tests (parametric) or Mann-Whitney U tests (nonparametric). Significance was set at *P* < .05. The study is reported in accordance with STrengthening the Reporting of OBservational studies in Epidemiology (STROBE).

Because of the small number of EPL ruptures, we performed a sensitivity analysis to determine the minimum detectable effect size for the principal comparisons (α = 0.05, two-sided; power 80%).

The study was approved by the Regional Ethics Committee at Umeå University, Sweden (Dnr 09-213). Written informed consent was obtained from all patients.

## Results

A total of 462 fractures in 449 patients aged 18–75 years were included for EPL rupture outcomes. EPL tendon status was systematically recorded in 419 fractures at 3 months and 373 at 12 months postinjury. Ten ruptures were identified overall (incidence of 2.4%, 95% CI: 1.3% to 4.3%), one acute and nine within 3 months, with none occurring thereafter.

There were seven ruptures in initially undisplaced/minimally displaced fractures versus three in displaced fractures (*P* = .06). Four ruptures occurred in surgically managed patients (three Kapandji pinning, one volar plate fixation) versus six in nonsurgically treated patients (closed reduction and cast immobilization; *P* = .99). We found no statistically significant differences between the EPL group and the remaining cohort in other studied parameters (Table).

**Table:** Baseline Characteristics and 12-Month Outcomes in Patients With and Without EPL Rupture After DRF

Variable	Cohort	Non-EPL	EPL	*P* Value (Non-EPL vs EPL)
No.	419	409	10	
Age (y), mean (SD)	56.5 (13.5)	56.5 (13.4)	55.3 (16.6)	.79
Sex (women/men)	321/98	313/96	8/2	.99
Dorsal comminution (yes/no)	297/122	290/119	7/3	.99
Treatment: nonsurgical/surgical	248/171	242/167	6/4	.99
Grip strength 12 mo, % of uninjured hand (mean, SD)	0.89 (0.24)	0.89 (0.25)	0.88 (0.14)	.90
Function with *Quick*DASH (mean, SD)	17 (18)	17 (18)	12 (9)	.39
Function with *Quick*DASH, median (IQR)	11 (2–26)	11 (2–27)	9 (5.5–14.8)	.76
Quality of life with EQ-5D (mean, SD)	0.92 (0.135)	0.93 (0.137)	0.80 (0.16)	.80
Quality of life with EQ-5D Median (IQR)	0.83 (0.8–1.00)	0.83 (0.82–1.00)	0.83 (0.81–0.84)	.44

IQR, interquartile range.

Of the 10 patients with EPL rupture, 7 underwent tendon transfer surgery, whereas 3 opted for nonsurgical management. The 12-month *Quick*DASH score was 11.67 in patients treated with tendon transfer and 11.50 in those managed nonsurgically (*P* = .98).

The sensitivity analysis indicated that the study could detect a difference of approximately 16 *Quick*DASH points, 0.22 in grip strength ratio, and 0.12 in EQ-5D index between patients with and without EPL rupture, and only a relative risk of 3.5 or greater for dichotomous predictors such as treatment modality or dorsal comminution.

## Discussion

This study confirms that EPL rupture remains a clinically important, though relatively uncommon, complication of DRFs, with an incidence of approximately 2.4% in this population. All ruptures occurred within three months of injury, consistent with previous observations that the critical period for delayed EPL rupture is typically between 3 and 12 weeks postfracture.^5^, ^6^, ^7^, ^8^, ^9^, ^10^ Importantly, these ruptures were not significantly associated with age, sex, or presence of dorsal comminution. There was no statistically significant difference in incidence between surgical and conservative treatment groups.

The pathogenesis of EPL rupture after DRF is multifactorial. Several mechanisms have been proposed, including vascular compromise at the hypovascular zone near Lister’s tubercle, increased tendon friction, mechanical attrition by fracture callus or hardware, hematoma-induced pressure, and direct injury from sharp bony fragments. In their register-based study of insurance claims for patient-reported maltreatment of DRFs in Sweden between 2011 and 2021, Thórdardóttir et al^17^ identified tendon injury among the most common complications. Although prominent dorsal screws after volar plating are a well-known cause, studies show that ruptures can occur even in the absence of surgical intervention or hardware, which underscores the complex interplay of anatomic and biomechanical factors.^8^, ^9^, ^10^ The narrow tendon sheath and the intact extensor retinaculum in minimally displaced fractures may create a compressive environment for the tendon, promoting late rupture.

Notably, this study could not identify any demographic or fracture-specific risk factor, nor a significant effect of treatment type or fracture displacement. This aligns with growing evidence that both nondisplaced and displaced fractures can result in rupture, and no protective effect of patient selection or specific surgical technique was demonstrated, although the analysis was not powered to exclude moderate effects. Although more ruptures occurred in undisplaced/minimally displaced fractures, the difference was not statistically significant (*P* = .06), paralleling other reports where rupture is often seen even after low-energy trauma or nondisplaced injury. However, the small number of patients in each group could also contribute to the lack of statistical significance of this parameter.

In our clinical practice, low-demand patients with EPL ruptures are treated nonsurgically, whereas other patients are managed with timely tendon reconstruction (usually by extensor indicis proprius transfer or similar procedures). In the present study, patients sustaining EPL rupture did not experience worse long-term outcomes in grip strength or patient-reported function and quality of life. Among the 10 patients with rupture, seven underwent tendon transfer, and three were managed nonsurgically, with similar 12-month *Quick*DASH scores. This comparison must be interpreted with caution: treatment was selected on clinical grounds, including functional demand, so the groups are not entirely comparable, and the numbers are far too small to establish equivalence. The observation is best summarized as indicating that acceptable outcomes were achieved in selected low-demand patients managed nonsurgically, not that the two strategies are equivalent.

The lack of strong, predictable risk factors for EPL rupture after DRF makes its prevention challenging. However, heightened clinical vigilance is warranted, particularly in the first 3 months postinjury. Routine clinical examination of active thumb extension at acute and follow-up visits, and judicious radiographic evaluation for hardware prominence in surgically managed patients, are prudent strategies. Early recognition allows for timely intervention and may prevent missed diagnosis and delayed repair, which are associated with worse outcomes. In their observational study, Kim et al^18^ analyzed preoperative computed tomography scans of patients with DRF and found that the sum of floating fat sign scores, ie, marrow fat leaking into the second and third compartments, was correlated with the rate of EPL rupture, helping to predict delayed cases.

This study has several limitations. Approximately 20% of the initially enrolled patients were lost to follow-up at 1 year, and the subgroup with EPL tendon rupture was relatively small.

Given the rarity of EPL rupture, this secondary analysis was underpowered to detect modest between-group differences. A sensitivity analysis indicated that the study could detect a difference of approximately 16 *Quick*DASH points, 0.22 in grip strength ratio, and 0.12 in EQ-5D index between patients with and without EPL rupture, and only a relative risk of 3.5 or greater for dichotomous predictors such as treatment modality or dorsal comminution. Smaller but potentially relevant differences therefore cannot be excluded, and the nonsignificant findings should be interpreted as an absence of evidence for large effects rather than as evidence of equivalence. This applies with particular force to the comparison between tendon transfer and nonsurgical management, in which only three patients were managed nonsurgically, and the minimum detectable difference in *Quick*DASH for this within-rupture subgroup (17–35 points) far exceeds the minimal clinically important difference. That comparison is therefore descriptive only.

Nevertheless, our findings offer a valuable real-world perspective on EPL rupture outcomes within a well-characterized prospective cohort, with descriptive insights into rupture timing and mechanisms that retain clinical relevance for hand surgeons irrespective of statistical power. Other key strengths include the prospective, multicenter design, standardized physical examinations for all participants, and the use of validated patient-reported outcome measures. These features enabled a robust, albeit exploratory, evaluation of this uncommon complication.

Overall, this cohort study highlights that EPL rupture is a clinically important yet uncommon complication following DRFs, occurring within the early postfracture period and without clearly predictable demographic, fracture-related, or treatment-related risk factors. These findings emphasize the importance of systematic early surveillance of active thumb extension, particularly during the first three months after injury, rather than relying solely on conventional risk factor assessment. In surgically managed patients, careful evaluation of implant positioning and potential hardware prominence remains essential. Although the number of EPL rupture cases was small, long-term grip strength, patient-reported function, and quality of life were comparable between patients with and without rupture, and acceptable outcomes were observed after tendon transfer, and also in the small number of selected low-demand patients in whom nonsurgical management was chosen. This supports an individualized treatment approach, with timely tendon reconstruction for appropriate patients and nonsurgical management considered in selected low-demand cases. Future adequately powered prospective studies are needed to clarify modifiable risk factors, improve preventive strategies, and optimize care pathways for this uncommon but important complication.

## Ethics declaration

Written informed consent to take part in the study and to publish the article has been obtained from all participants or their legal representatives. The privacy rights of participants have been observed.

This study was performed in compliance with relevant laws, regulatory frameworks and guidelines where the research took place. This study was approved by the Umeå University. (Approval No. D nr 09-213, 2009-1419-31????????? ????????????)

## Conflicts of Interest

No benefits in any form have been received or will be received related directly to this article.

## Disclosures Human subjects

Informed consent for treatment and open access publication was obtained or waived by all participants in this study. Institutional Review Board (IRB) or ethics committee issued approval Dnr 09-213. The study was approved by the Regional Ethics Committee at Umeå University, Sweden (Dnr 09-213). Written informed consent was obtained from all patients.
